# Service robots on their way? First steps of an interdisciplinary technology assessment

**DOI:** 10.1007/s10202-012-0112-7

**Published:** 2012-11-21

**Authors:** Michael Decker, Ulrike Henckel

**Affiliations:** 1 Institute for Technology Assessment and Systems Analysis, Karlsruhe Institute of Technology, Karlstrasse 11, 76133 Karlsruhe, Germany; 2 Europäische Akademie GmbH, Wilhelmstraße 56, 53474 Bad Neuenahr-Ahrweiler, Germany

Interdisciplinary research calls together different scientific disciplines in order to answer a research question which cannot be answered by an individual discipline alone. Technology Assessment (TA) is a problem-oriented approach (Bechmann and Frederichs 1996) dealing with the non-technical aspects of technology development, in order to gain knowledge about the (un-)intended consequences, the (un-)desired impacts, the main and side-effects and the chances and risks of (new) technologies. Moreover, by applying TA, scientists can develop potential solutions to solve societal or political problems related to, for example, the “grand challenges” such as “feeding 10 billion people,” demographic change, global health. These societal problems need to be reframed or transformed into research questions to be dealt with by interdisciplinary research. Which scientific disciplines are invited to participate in an interdisciplinary research project is defined with respect to these research questions, namely those which are identified as to be relevant to answer them.

Therefore, framing the problems and developing research questions out of it becomes the key for any interdisciplinary project (Decker 2001). For the interdisciplinary TA project on service robotics, to which the papers of this focus contribute, the problem setting and the identification of the relevant disciplines were described elsewhere (Decker et al. 2011; Decker 2012). Since the project aims for a TA of service robotics on a general level (even though referring to case studies for illustration), the relevant disciplines include economics, ethics, jurisprudence and psychology of work. While the first three are traditionally part of an interdisciplinary TA, the latter one is crucial in the context of replacing human beings in their service work environments.

The contributions to this focus can be described as “interdisciplinary-enriched disciplinary perspectives” on the problem at stake. It is, thus, taken into account that interdisciplinary projects typically start with disciplinary perspectives, often referred to as “multidisciplinary starting phase.” Here, the different representatives of the scientific disciplines present their point of view to the other experts in the project group and get the respective feedbacks during the interdisciplinary discussion. By considering these responses for the further development of their disciplinary perspective, their papers get “interdisciplinary enriched.” The questions during this feedback process very often cross disciplinary boundaries: Each discipline gets questions from the other research areas involved and answers them in the next version of their paper.

This “game” of cross-disciplinary questioning and answering is the core of interdisciplinary research and therefore needs—as, for example, the problem framing, too—an additional advice (or a quality control) by external experts, that is, experts not involved in the project group. These are explicitly invited to check both whether their “disciplinary colleague” asked the relevant questions to the other disciplines and whether he or she included sensible answers to the questions posed to their own discipline in the paper. This advice was given during the midterm meeting in July 2012.^1^ During the second half of the project, these essays are currently developed to a TA report in common authorship of all group members in order to reach an interdisciplinary text which will serve as an argumentation basis for recommendations given to policy makers and the general public.

Since robotics in general and service robotics in particular have been investigated during the last years by both technology assessors and Science and Technology Studies (STS) experts, Decker illustrates in the first paper the state of the art in these fields. First recommendations to act were developed by different TA groups especially focusing on autonomous systems. These studies also developed first criteria on how service robots should look like. The review focuses on first results from empirical studies and presents first results with respect to acceptance of service robots.

If one describes service robots as to be autonomous, one causes special attention by both the ethical resp. the anthropological and the legal reflection. Main juridical problems are issues of responsibility and liability: Who is liable, if, for example, a learning robot gets something “wrong” and causes damage? In the contribution on legal issues, Dreier and Spiecker genannt Döhmann explain that the emergent use of service robots in social situations causes a lot of legal problems without having to refer to concrete norms and particular court rulings. They give, starting with some words about the general ideas about law as a means to regulate and govern technology, an overview over some of the urgent questions that arise for the legal field by the far-reaching use of service robotics. As a result, they claim that in order to be able to establish standards for negligence, certain basic safety rules must be in place in private and freely accessible public space which the operators of service robots have to comply with.

The economic perspective needs to start with a reflection on the notion ‘service’ itself, as the so-called tertiary sector has undergone a shift from traditional to novel types of services. The latter are, according to Ott in this volume, characterized by strong knowledge intensity, mostly due to their linkages to certain technologies. From an ecological point of view, dealing with service robots on a general level is not feasible, since a strong contextualization needs to be taken into account. However, Ott expects a positive net labor market effect as she assumes that service robots are going to end up in overall job creation that goes along with increasing skill standards of the employees involved.

Here, the psychological/work science comes into play. With a special focus on man–machine interactions, Fischer develops three design perspectives which should be taken into consideration for establishing criteria of usability. It is crucial to exactly identify which tasks in these interactions are “handed over” to the robot and which remain by the human. In contrast to applications of industrial robots, service tasks are characterized by a close costumer–client relation, often even including direct contact, which results in a number of implications in terms of design and utilization of service robots. Moreover, the author points to the fact that usability criteria must be augmented, firstly, by characteristics covering the hardware and software technology components of the robot and, secondly, by criteria that pertain to the “relationship quality” of the robot.

The technological perspective is also included in this TA project but not presented in this focus, since the midterm results of the technological perspective mainly describe the technical state of the art and present a context-related description of the case studies. A general overview on service robotics can be found in Schraft and Schmierer (1998), Schraft et al. (2004). Next steps of the interdisciplinary TA project will be to integrate the disciplinary arguments developed so far into interdisciplinary argumentation chains grounding the recommendations. These results are expected to be published in summer 2013.

## Footnotes

1 All authors of this focus are members of the project group “Technology Assessment of Service Robots”, organized by the Europäische Akademie zur Erforschung von Folgen wissenschaftlich-technischer Entwicklungen Bad Neuenahr-Ahrweiler GmbH (www.ea-aw.de). The group would like to thank Mrs. Blechschmitt, Dr. Diego Compagna, Professor Dr. Wolfgang Gessner, Dr. Martin Hägele, Mr. Severin Löffler, Professor Dr. Ursula Weidenfels and Professor Klaus Wiegerling who evaluated the work in progress during the midterm meeting.

## References

[CR1] Bechmann G, Frederichs G, Bechmann G (1996). Problemorientierte Forschung: Zwischen Politik und Wissenschaft. Praxisfelder der Technikfolgenforschung. Konzepte, Methoden, Optionen.

[CR2] Decker M (2001). Interdisciplinarity in technology assessment. Implementation and its chances and limits.

[CR3] Decker M, Decker M, Gutmann M (2012). Technology of service robotics. Preliminary thoughts guided by case studies. Robo- and informationsethics. Some fundamentals.

[CR4] Decker M, Dillmann R, Dreier T, Fischer M, Gutmann M, Ott I, Spiecker genannt Döhmann I (2011). Service robotics: do you know your new companion? Framing an interdisciplinary technology assessment. Poiesis Prax.

[CR5] Schraft RD, Schmierer G (1998). Serviceroboter—Produkte, Szenarien, Visionen.

[CR6] Schraft RD, Hägele M, Wegener K (2004). Service Roboter Visionen.

